# Supplementary material for: An analgesic pathway from parvocellular oxytocin neurons to the periaqueductal gray in rats
Source: Nat Commun. 2023 Feb 24;14:1066. doi: 10.1038/s41467-023-36641-7 (PMC9958129; doi:10.1038/s41467-023-36641-7)
Supplement: Supplementary file 1 — Supplementary Information [file 41467_2023_36641_MOESM1_ESM.pdf]

An analgesic pathway from parvocellular oxytocin neurons to the periaqueductal gray in rats

Supplementary Information

Mai Iwasaki[1,#], Arthur Lefevre[1,2,+,#], Ferdinand Althammer[2,3,4,#], Etienne Clauss Creusot[1,#], Olga Łąpieś[1], Hugues Petitjean[1], Louis Hilfiger[1], Damien Kerspern[1], Meggane Melchior[1], Stephanie Küppers[2], Quirin Krablicher[2], Ryan Patwell[2], Alan Kania[2], Tim Gruber[5], Matthew K. Kirchner[3], Moritz Wimmer[4], Henning Fröhlich[4], Laura Dötsch[4], Jonas Schimmer[2], Sabine C, Herpertz[6], Beate Ditzen[7,8], Christian P. Schaaf[4,8], Kai Schönig[9], Dusan Bartsch[9], Anna Gugula[10], Aleksandra Trenk[10], Anna Blasiak[10], Javier E. Stern[3], Pascal Darbon[1], Valery Grinevich[2,3,§,*], Alexandre Charlet[1,§,*]

[1] Centre National de la Recherche Scientifique and University of Strasbourg, Institute of Cellular and Integrative Neuroscience, 67000 Strasbourg, France
[2] Department of Neuropeptide Research in Psychiatry, Central Institute of Mental Health, University of Heidelberg, Mannheim 68159, Germany
[3] Center for Neuroinflammation and Cardiometabolic Diseases, Georgia State University, Atlanta, GA, USA
[4] Institute of Human Genetics, University Hospital Heidelberg, Heidelberg, Germany
[5] Van Andel Institute, Grand Rapids, MI, USA
[6] Department of General Psychiatry, Center of Psychosocial Medicine, University of Heidelberg, 69115, Heidelberg, Germany
[7] Institute of Medical Psychology, Heidelberg University Hospital, 69115, Heidelberg, Germany
[8] Ruprecht-Karls University Heidelberg, Heidelberg, Germany
[9] Department of Molecular Biology, Central Institute of Mental Health, University of Heidelberg, Mannheim 68159, Germany
[10] Department of Neurophysiology and Chronobiology, Institute of Zoology and Biomedical Research, Faculty of Biology, Jagiellonian University, Krakow, 30-387, Poland
+ Present address: Cortical Systems and Behavior Laboratory, University of California, San Diego, La Jolla, CA 92093, USA

# These authors contributed equally
§ These authors jointly supervised this work

Supplementary Figure 1

a, Schema of the generation of knock-in OTR-Cre rats.

b, As a further control, DNAs of Cre positive animals were used for detection of homologous recombination at the OT locus. For this purpose, the targeted region was amplified by PCR using the Q5 polymerase (NEB) with 100 -200ng of genomic DNA as template. Primers were selected which bind up- and downstream of the insertion site (outside of the homology arms) and were each combined with a primer located within the IRES2-Cre construct. We used for the 5' insertion site the primers OT upper_check (5′ CTCCCAGGGAAGATCTGTACC 3′) together with IRES2_rev (5′ GCTTCGGCCAGTAACGTTAGG 3′) and for the 3' site the primers CRISPR_bGH_for (5' gacaatagcaggcatgctgg3') together with OT_rev_hom (5' GGGTTTTCCCAGAACTCAGC 3').

c, Injection schema for the labelling of OTR neurons in the OTR-Cre rats. The $rAAV_{1/2}$-EF1α-DIO-GFP is injected into the PAG.

d-f, Images showing GFP (green) and DAPI (blue) staining, at various distances from Bregma, in the vlPAG of OTR-Cre rats injected with the EF1α-DIO-GFP rAAV. Scale bar = 100µm. Aq = Aqueduct.

h-i, TGOT-induced increase of AP frequency of GFP-negative neurons in the PAG of female rats. **h,** Example traces from a GFP-negative cell under baseline, TGOT application, and wash out conditions. **i,** Quantification of TGOT effect on AP frequency of GFP-negative neurons (Baseline 0.119 ± 0.046 Hz vs TGOT 0.108 ± 0.049 Hz vs Wash 0.122 ± 0.064 Hz; Friedman's test = 1.6, p = 0.48, n_{cells} = 9).

j-m, TGOT-induced change in first spike latency (FSL) of GFP-negative neurons in the PAG of female rats. **j**. Representative evoked currents in a GFP-negative neuron in response to a square current step (50 pA; 800 ms) in baseline (black line) and oxytocin (blue line) conditions. **k,** FSL quantification for GFP-negative neurons (OTR^-) during a square current step: no differences were found between the baseline condition (31.95 ± 9.44 ms) and after the TGOT incubation 37.08 ± 14.89 ms, non significative (n.s.) p = 0.788 (paired two-tailed t test, n_{cells} = 7). **l,** Proportion of neuron after TGOT incubation with a decrease of the FSL (n = 1/7, blue); an increase (n = 2/7, red) or without a variation of ± 10 ms (no effect, gray (n = 4/7). **m,** Quantification of TGOT effect on AP frequency of GFP-negative neurons during a square current step (Baseline 7 ± 2.012 spikes vs TGOT 7.857 ± 2.604 spikes; Wilcoxon signed-rank test = 6, p = 0.6845 (two-sided), n_{cells} = 7.

Data are represented as mean ± sem and as individual paired points. Source data are provided as a Source Data file.

Figure S1

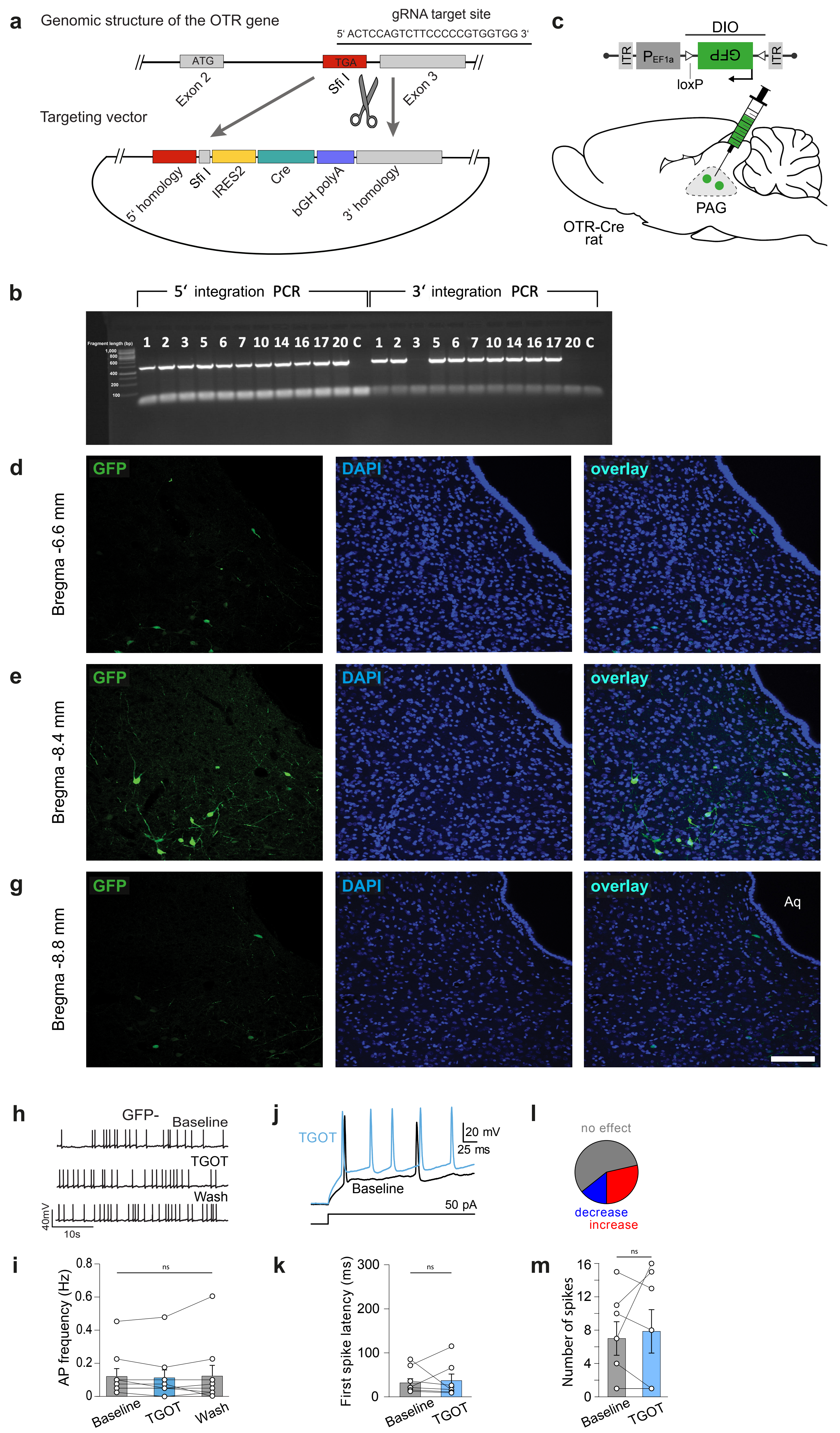

a
Genomic structure of the OTR gene
gRNA target site
5' ACTCCAGTCTTCCCCCGTGGTGG 3'
ATG
TGA
Exon 2
Sfi I
Exon 3
Targeting vector
5' homology
Sfi I
IRES2
Cre
bGH polyA
3' homology
c
DIO
ITR
PEF1a
GFP
loxP
PAG
OTR-Cre rat
b
5' integration PCR
3' integration PCR
1 2 3 5 6 7 10 14 16 17 20 C
1 2 3 5 6 7 10 14 16 17 20 C
d
Bregma -6.6 mm
GFP
DAPI
overlay
e
Bregma -8.4 mm
g
Bregma -8.8 mm
Aq
h
GFP-
Baseline
TGOT
Wash
40mV
10s
j
TGOT
Baseline
20 mV
25 ms
50 pA
l
no effect
decrease
increase
i
AP frequency (Hz)
ns
Baseline
TGOT
Wash
k
First spike latency (ms)
m
Number of spikes

Supplementary Figure 2

a, RNAscope for OTR and Cre mRNA probes. Enlarged insets highlight co-localization of OTR and Cre probe signals. Aq=aqueduct, DR=dorsal raphe nucleus, vlPAG=ventrolateral periaqueductal gray.

b, Quantification of OTR/Cre and Cre/OTR probe signals from n = 3 rats (3 sections each). Results are expressed as the mean ± SEM and the individual points of each conditions are represented as white circle.

c, Confocal images show the vlPAG of a OTR-IRES-Cre rat injected with a cre-dependent rAAV expressing GFP that was counterstained with a cre antibody. High magnification images highlight co-localization of cre protein and GFP signals.

d, Quantification of cre+ GFP-expressing cells in vlPAG (GFP/Cre). Results are expressed as the mean ± SEM and the individual points of each conditions are represented as white circle.

e, Uncut western blot of WT and OTR-IRES-Cre (KI) rats using a cre antibody.

f, Enlarged inset shows presence of specific cre band (35kDa) in a KI, but not WT rat.

Source data are provided as a Source Data file.

Figure S2

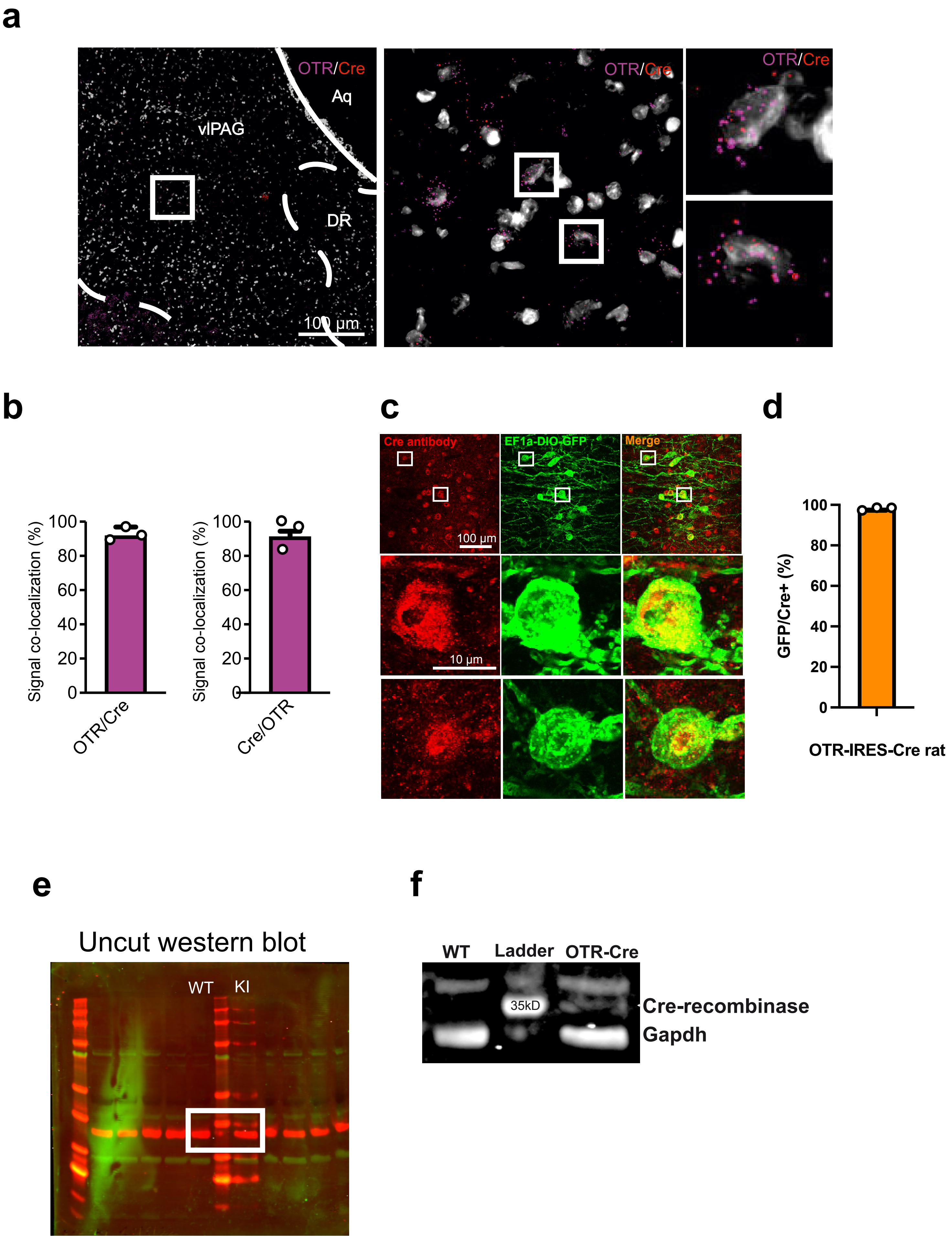

a
OTR/Cre
Aq
vlPAG
DR
100 µm
OTR/Cre
OTR/Cre
b
Signal co-localization (%)
100
80
60
40
20
0
OTR/Cre
Signal co-localization (%)
100
80
60
40
20
0
Cre/OTR
c
Cre antibody
EF1a-DIO-GFP
Merge
100 µm
10 µm
d
GFP/Cre+ (%)
100
80
60
40
20
0
OTR-IRES-Cre rat
e
Uncut western blot
WT
KI
f
WT
Ladder
OTR-Cre
35kD
Cre-recombinase
Gapdh

Supplementary Figure 3

Schematic depiction of experimental procedures. WT and OTR-IRES-Cre rats were subjected to stereotaxic injection of various viral vectors at 10-12 weeks of age. Rats were then allocated to either neuroanatomical or physiological studies and processed accordingly. Images were created using BioRender.

Figure S3

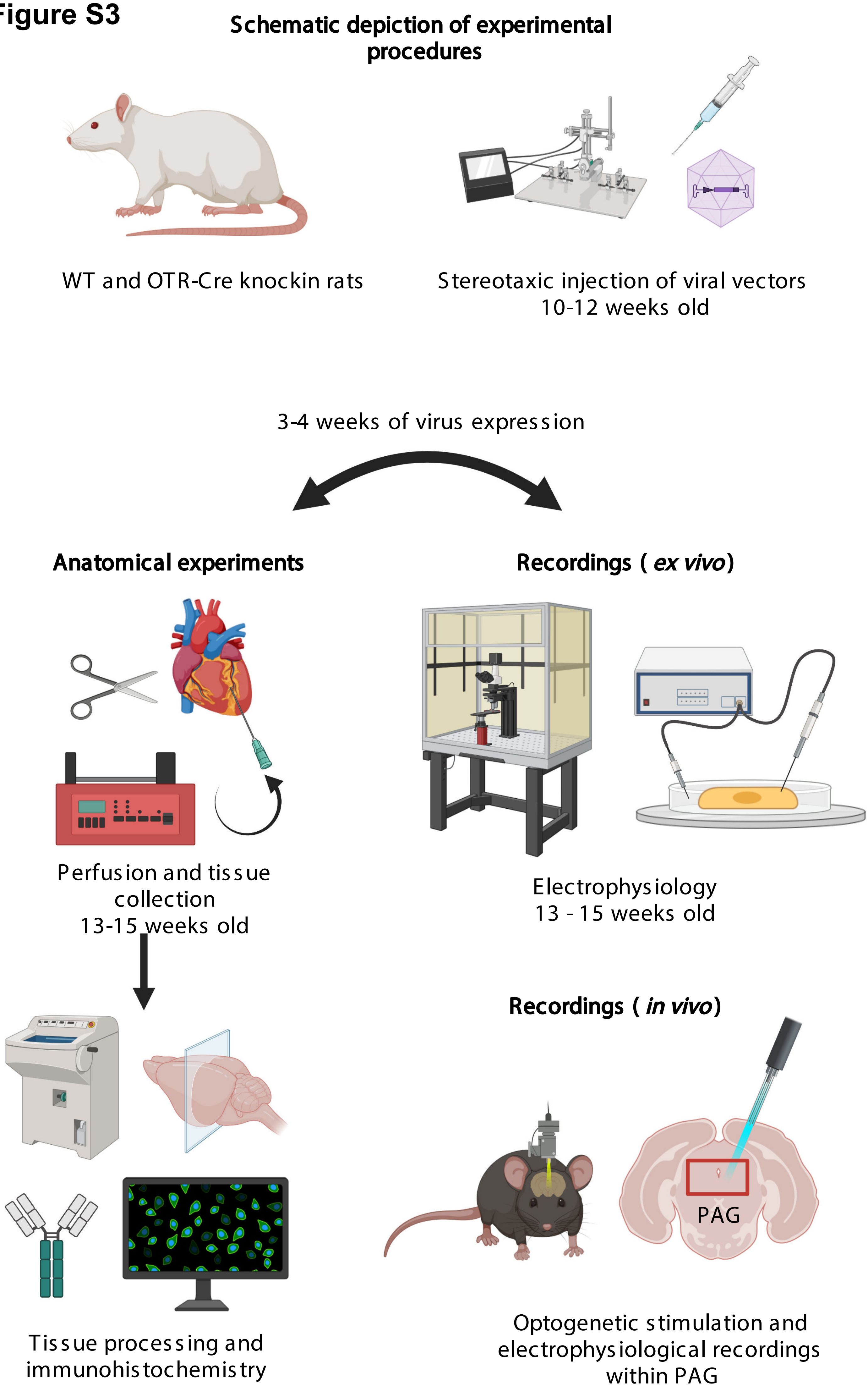

Schematic depiction of experimental procedures
WT and OTR-Cre knockin rats
Stereotaxic injection of viral vectors
10-12 weeks old
3-4 weeks of virus expression
Anatomical experiments
Recordings (ex vivo)
Perfusion and tissue collection
13-15 weeks old
Electrophysiology
13 - 15 weeks old
Recordings (in vivo)
PAG
Tissue processing and immunohistochemistry
Optogenetic stimulation and electrophysiological recordings within PAG

Supplementary Figure 4

a, Unilateral stereotaxic injection of WT rats (n=3 male and n=3 female) into the SON and PVN using $rAAV_{1/2}$-EF1α-mCherry or $rAAV_{1/2}$-EF1α-GFP, respectively.

b, Verification of injection sites and correct expressing of viral fluorescent proteins in the respective nuclei.

c, Analysis of OTergic fiber laterality within the vlPAG of male and female rats and various Brema levels. Note that OT fibers are prominently labeled in both hemispheres (i.e. left and right vlPAG) following unilateral injections into the PVN.

Figure S4

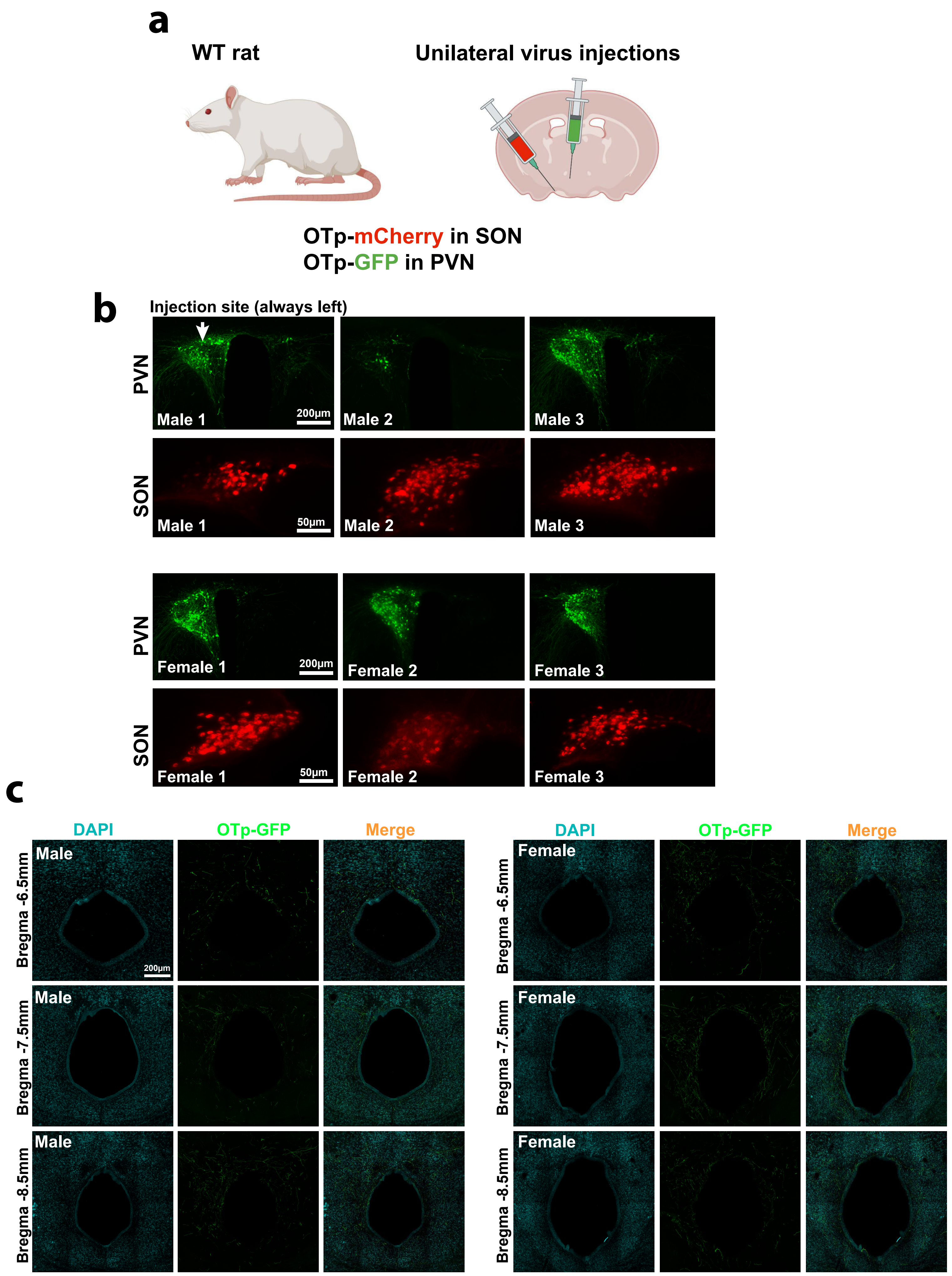
a
WT rat
Unilateral virus injections
OTp-mCherry in SON
OTp-GFP in PVN
b
Injection site (always left)
PVN
SON
Male 1
Male 2
Male 3
Female 1
Female 2
Female 3
200μm
50μm
c
DAPI
OTp-GFP
Merge
Male
Female
Bregma -6.5mm
Bregma -7.5mm
Bregma -8.5mm
200μm

Supplementary Figure 5

a-b, Distinction of parvocellular (parvOT) and magnocellular (magnOT) OT-neuron populations. **a,** Schema for the identification of magnOT and parvOT neurons: injection of green retrobeads into the PAG, staining for OT (red) and central application of fluorogold (FG, blue), which labels only magnOT neurons. **b,** Image of retrolabeled neurons containing fluorescent beads (green) as well as staining for Fluorogold (blue) and OT (red). White arrows indicate the co-localization of OT and FG (magnOT neuron) and the white asterisk indicates the co-localization of retrobeads and OT. Scale bars = 25 µm. n = 3 female rats.

c-f, Retrograde labelling of projections from PVN OT-neurons to the vlPAG and SC. **c,** Schema of the experimental design with injection site of green retrobeads in the vlPAG (**d**) and red retrobeads in the SC (L5, **e**). Scale bars = 200 µm. **f,** Image of PVN OT-neurons (blue) projecting either to the vlPAG (green, white asterisk) or to the SC (red, white arrow). No OT neuron containing both red and green retrobeads was found. Aq = Aqueduct. 3v = third ventricle. Scale bar = 50µm. n = 4 female rats.

g-h, PAG-projecting PVN OT-neurons from Figure 2B do not target the SC or SON. **g,** Schema of viral injection showing injection of $rAAV_{1/2}$-OTp-DIO-GFP in the PVN and rAAV-CAV2-Cre in the vlPAG of WT rats. **h,** Images of slices from the spinal cord at different levels (cervical, C3; thoracic, T7; lumbar, L4) and from the SON. No GFP fibers were found. Scale bars = 200 µm. n = 2 female rats.

i-j, SON-projecting PVN OT-neurons target the SC but not the vlPAG. **i,** Schema of viral injection showing injection of $rAAV_{1/2}$-OTp-DIO-GFP in the PVN and rAAV-CAV2-Cre in the SON of WT rats. **j, Left** Images of PVN OT-neurons (red) expressing GFP (green) projecting fibers to the SC (L5, NeuN in red, scale bars = 100 µm). **Right** Image of the vlPAG showing no GFP positive fibers. Scale bar = 100 µm. n = 4 female rats.

k-l, SC-projecting PVN OT-neurons target the SON but not the vlPAG. **k,** Schema of viral injection showing injection of $rAAV_{1/2}$-OTp-DIO-GFP in the PVN and rAAV-CAV2-Cre in the SC of WT rats. **l Left** Images of PVN (scale bars = 40 µm) OT-neurons (red) expressing GFP (green) projecting fibers to the SON (scale bar = 100 µm). opt = optic tract. **Right** Image of the vlPAG showing no GFP positive fibers. Aq = aqueduct. Scale bar = 100 µm. n = 4 female rats.

Figure S5

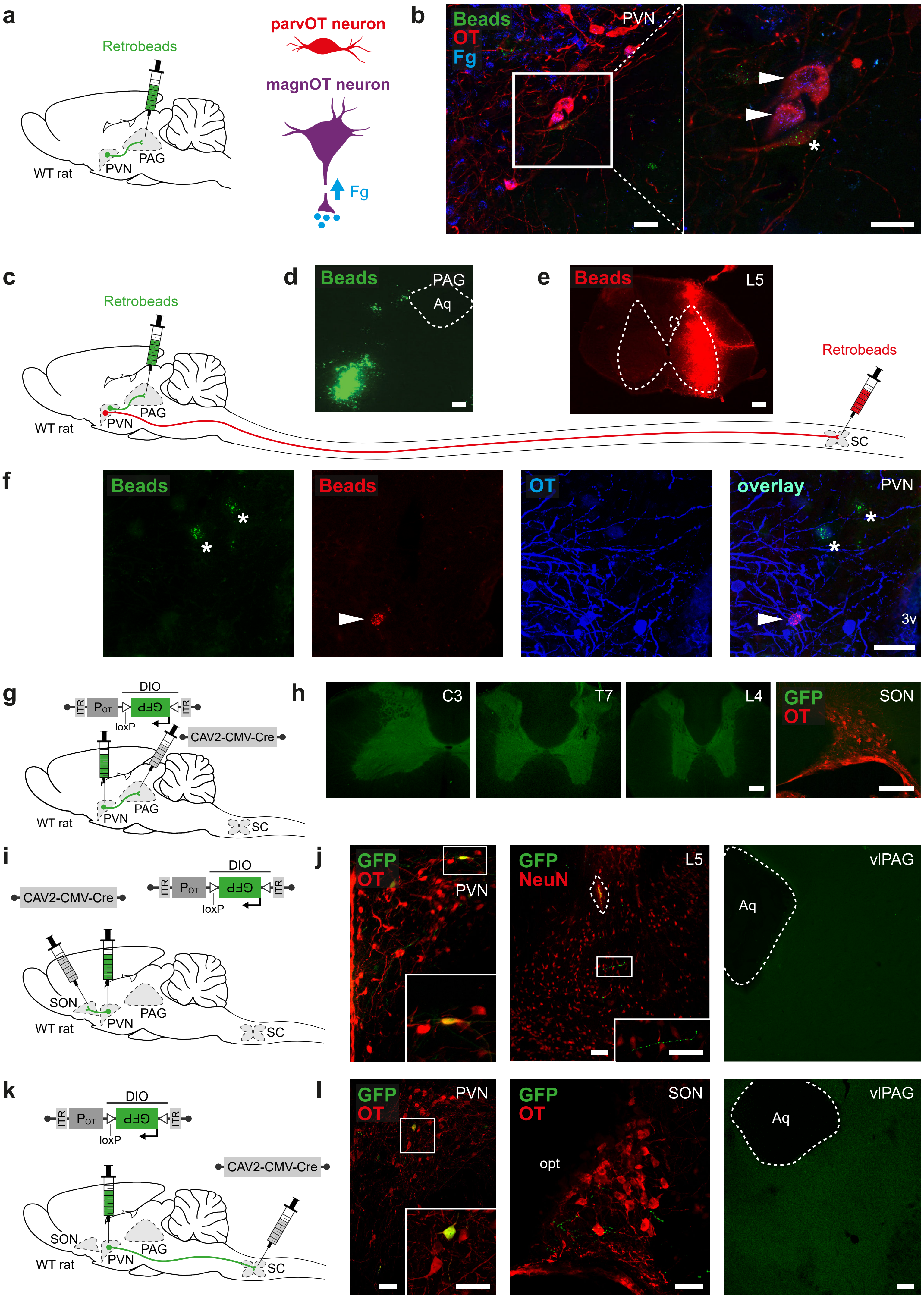
a
Retrobeads
PAG
PVN
WT rat
parvOT neuron
magnOT neuron
Fg
b
Beads
OT
Fg
PVN
c
Retrobeads
PAG
PVN
WT rat
d
Beads
PAG
Aq
e
Beads
L5
Retrobeads
SC
f
Beads
Beads
OT
overlay
PVN
3v
g
DIO
ITR
POT
GFP
ITR
loxP
CAV2-CMV-Cre
PAG
PVN
WT rat
SC
h
C3
T7
L4
GFP
OT
SON
i
CAV2-CMV-Cre
DIO
ITR
POT
GFP
ITR
loxP
SON
PAG
PVN
WT rat
SC
j
GFP
OT
PVN
GFP
NeuN
L5
vlPAG
Aq
k
DIO
ITR
POT
GFP
ITR
loxP
CAV2-CMV-Cre
SON
PAG
PVN
WT rat
SC
l
GFP
OT
PVN
GFP
OT
SON
opt
vlPAG
Aq

Supplementary Figure 6

Scheme depicting the respective projections of the two different neuronal populations of parvOT and their projections to spinal cord (Eliava et al., 2016) and to vlPAG (present manuscript). Given that the oxytocinergic innervation of the PAG appears to be higher by roughly 2-3-fold, we speculate that approximately 50-70 parvocellular OT cells might project to the vlPAG compared to the approximately 30 parvocellular OT cells that we previously reported for the spinal cord.

Figure S6

Distribution map of distinct parvocellular oxytocinergic projections from PVN to PAG and SC

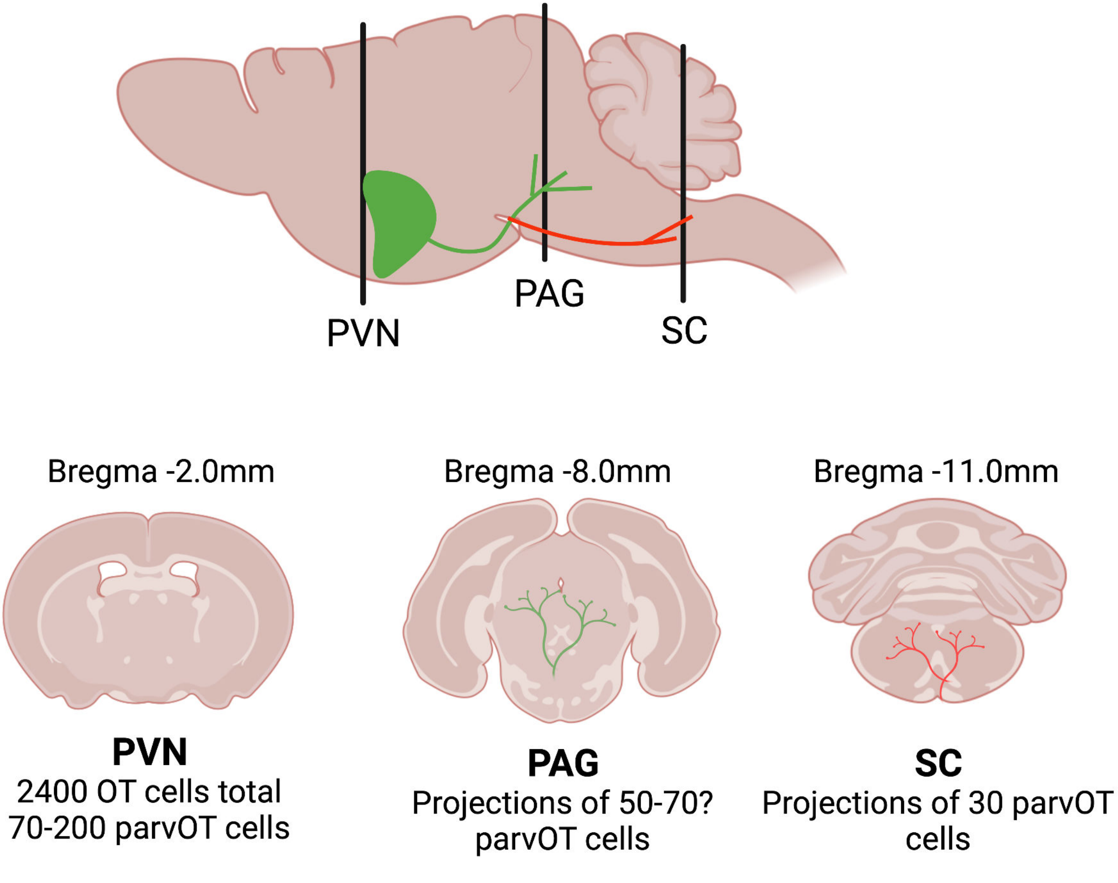

Supplementary Figure 7

a, Pipeline for Imaris-assisted 3D reconstruction based on raw IHC images.

b-c, Confocal images showing OT fibers and OTR-positive neurons in the vlPAG. **b,** Images of OTR neurons (green), OT fibers (magenta), vGluT2 (red) and DAPI (blue). Scale bar = 100 µm. **c,** Overlay. Scale bar = 100 µm.

d, Automated quantification of the number of SYN positive voxels in GFP-positive and negative neurons reveals no difference in overall synaptic input. n = 4 rats, 8 images per animal, p=0.6689, two-tailed student t-test. Results are expressed as the mean ± SEM and the individual points of each conditions are represented as white circle.

e, Analysis of somatic volume reveals no difference between OTR-positive (n = 496) and OTR-negative (n = 3840) soma sizes. n = 4 rats, 8 images per animal, p = 0.8104, two-tailed student t-test. Results are expressed as the mean ± SEM and the individual points of each conditions are represented as white circle.

f, Quantification of Syn+ OTergic fibers in vlPAG at various Bregma levels.
Individual data and statistics are presented in Source Data File. Results are expressed as the mean ± SEM and the individual points of each conditions are represented as white circle.

Source data are provided as a Source Data file.

Figure S7

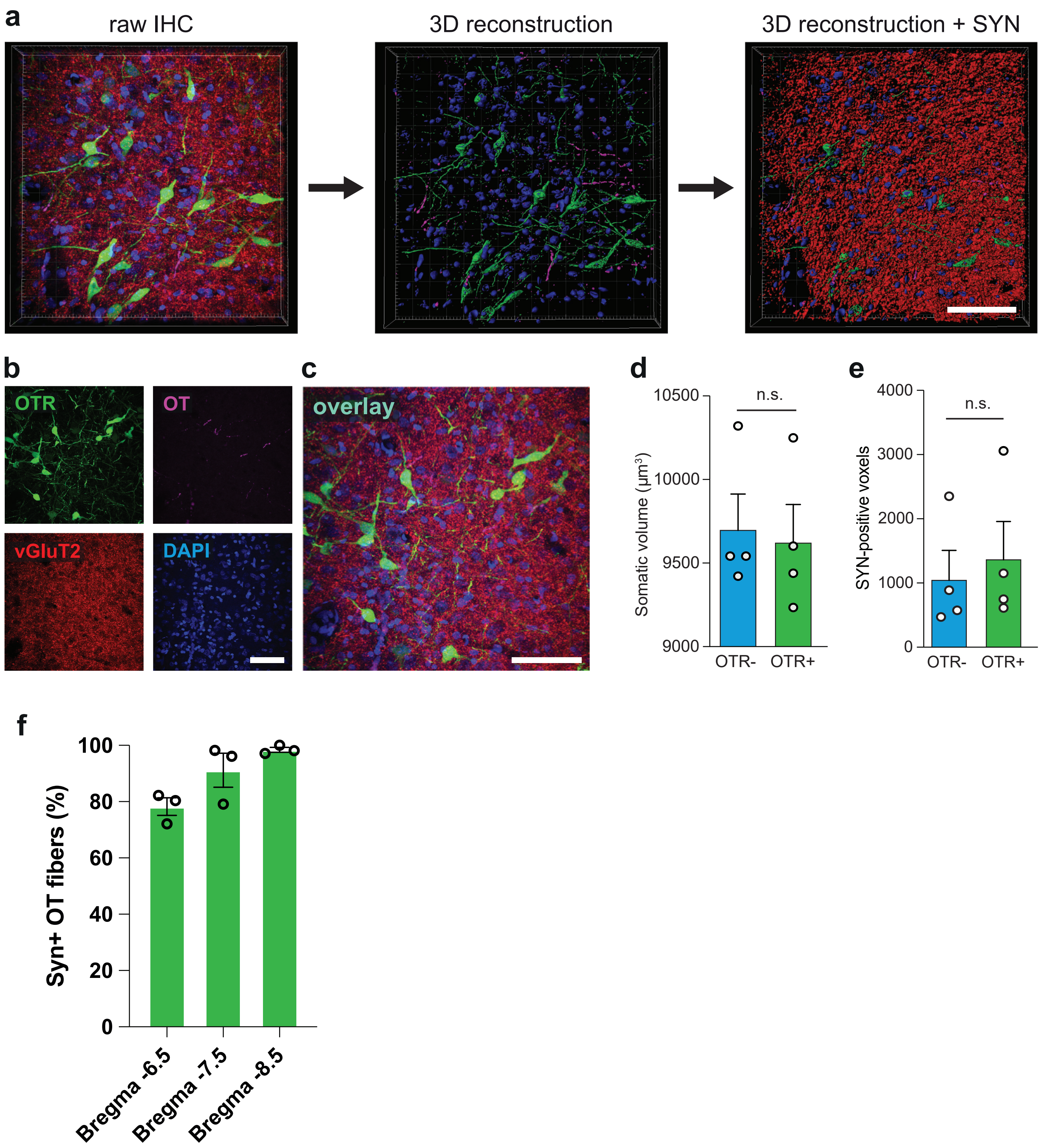
a
raw IHC
3D reconstruction
3D reconstruction + SYN
b
OTR
OT
vGluT2
DAPI
c
overlay
d
n.s.
Somatic volume (μm³)
10500
10000
9500
9000
OTR-
OTR+
e
n.s.
SYN-positive voxels
4000
3000
2000
1000
0
OTR-
OTR+
f
Syn+ OT fibers (%)
100
80
60
40
20
0
Bregma -6.5
Bregma -7.5
Bregma -8.5

Supplementary Figure 8

a, Visualization of the last step of spike sorting- superparamagnetic clustering (ascription of spikes to their corresponding neurons). This step is preceded by 1) automatic spike detection based on amplitude threshold and feature extraction – wavelets coefficients. From left to right: superimposed clusters, cluster separation and unassigned spikes based on spike shapes

b, Spike shapes represented in each channel of the tetrode.

c, Cluster 3 (from B) detailed profile, depicting (from left to right, and up to down) spikes' waveforms, baseline & peak voltages, spikes waveform variability over time, spikes parameters, stability presented as spikes count/time, activity during interspikes intervals of different length, density plots, waveform of selected spikes, PSTH, waveforms detected around an event and spikes distribution.

d, Onset, peak and offset of BL-induced excitation in the vlPAG. Onset is defined as the first time point when the spike rate exceeded the threshold (BS_{mean} + $4BS_{SD}$); BS_{mean} represents the mean firing rate of the baseline period, and BS_{SE} is the standard deviation of firing rate during the baseline period. Offset is defined as the last time point when the spike rate fell below the threshold for more than 20 s during the 300 s following BL (n_{cells} = 21).

e, Number of active cells in each single second. "Active" defined by a spike rate above the threshold (BS_{mean} + $4BS_{SD}$) (n = 21).

f, Image showing a representative recording site in the vlPAG. Dotted line indicates placement of the optic fiber. Aq = aqueduct. Scale bar = 100 µm.

Source data are provided as a Source Data file.

Figure S8

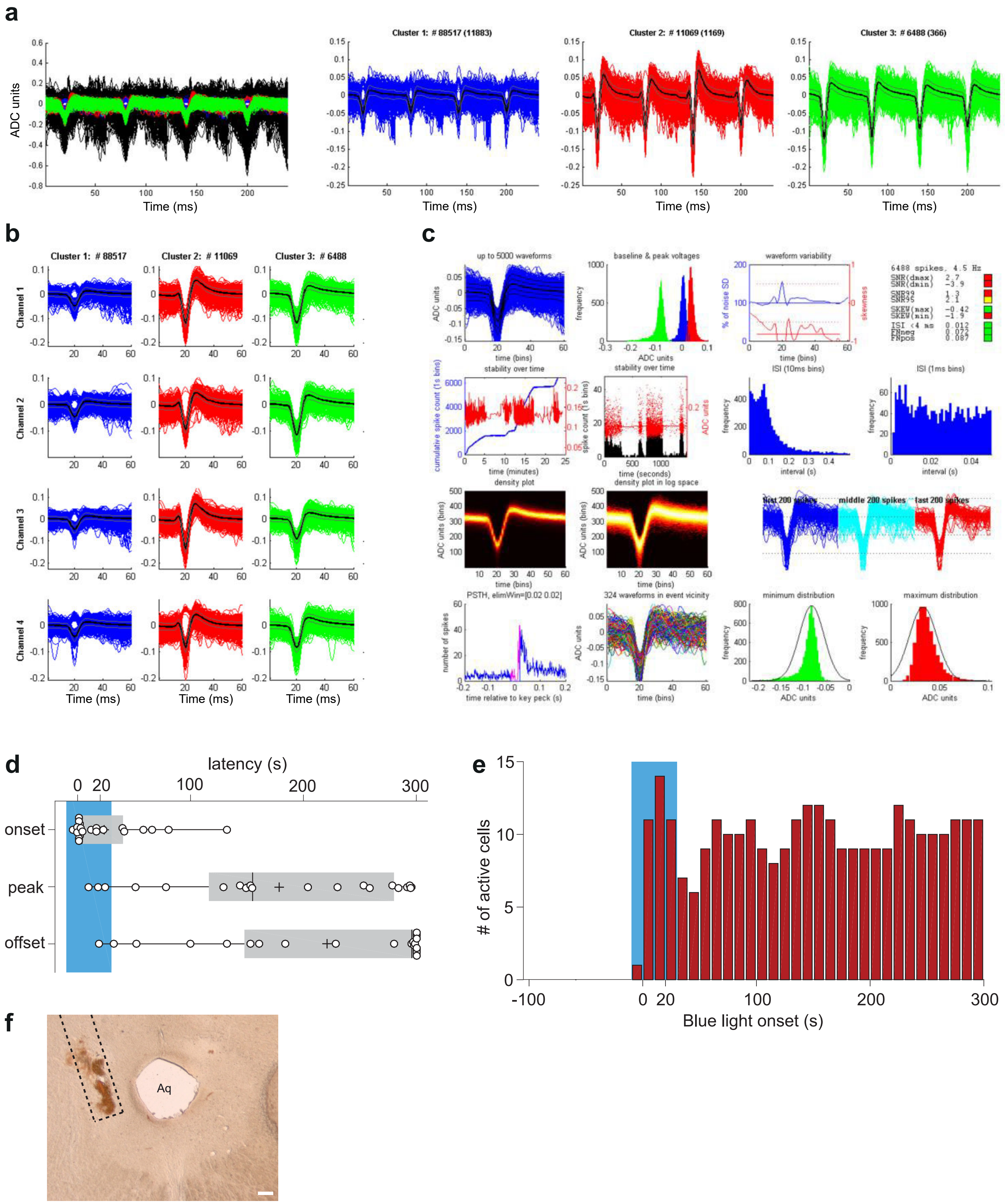
a
ADC units
Time (ms)
Cluster 1: # 88517 (11883)
Cluster 2: # 11069 (1169)
Cluster 3: # 6488 (366)
b
Cluster 1: # 88517
Cluster 2: # 11069
Cluster 3: # 6488
Channel 1
Channel 2
Channel 3
Channel 4
c
up to 5000 waveforms
baseline & peak voltages
waveform variability
6488 spikes, 4.5 Hz
stability over time
ISI (10ms bins)
ISI (1ms bins)
density plot
density plot in log space
first 200 spikes
middle 200 spikes
last 200 spikes
PSTH, elimWin=[0.02 0.02]
324 waveforms in event vicinity
minimum distribution
maximum distribution
time relative to key peck (s)
d
latency (s)
onset
peak
offset
e
# of active cells
Blue light onset (s)
f
Aq

Supplementary Figure 9

a, Scheme of electrophysiological recordings from SC in female rats.

b, Box plot showing maximum activity, minimum activity, and half activity latencies. Whiskers indicate the minimum and maximum latency. Colored box shows the range between 1st and 3rd quantile, median is depicted as vertical line. Unpaired Wilcoxon rank sum tests were performed. WT n=8, ChR2 n=15 (1st quartile, median, 3rd quantile); Latency to reach the maximum WU (s) [WT:(26.00, 31.50, 46.00), ChR2: (26.25, 35.00, 55.75)] not significant (p = 0.85); latency to reach the half reduction of WU (s) [WT: (75.50, 96.50, 163.00), ChR2 (64.25, 83.00, 125.75)] not significant (p = 0.32); or latency to reach the maximum reduction of WU (s) [WT (183.00, 209.00, 263.00), ChR2 (198.50, 250, 269.75)] not significant (p=0.50).

c-h, vlPAG BL effect on the spike rate of WDR's Aδ -fiber discharge. **c,** Mean time course observed after vlPAG BL in control rats (gray, n_{cells} = 8) and OT ChR2-expressing rats (blue, n_{cells} = 14). Left and right panels show two consecutive recordings separated by 300 s. Line shadows represent SEM. **d,** Percentage of reduction expressed as the minimum level activity observed after a wind-up plateau phase, 140 – 180 s; Unpaired Wilcoxon rank sum test (two-sided); Ctrl (n_{cells} = 8) 34.08 ± 4.73 vs ChR2 (n_{cells} = 14) 51.26 ± 4.1%, U = 27, p = 0.0337 and **e** 570 – 600 s after BL onset. Unpaired Wilcoxon rank sum test (two-sided); CTRL (n_{cells} = 8) 19.26 ± 16.88 vs ChR2 (n_{cells} = 14) 33.88 ± 5.12 %, U = 37, p = 0.2119. **f,** Mean time course observed after vlPAG BL in OT ChR2-expressing rats (blue, n_{cells} = 6), and in the same recordings after dOVT injection in the vlPAG (red, n_{cells} = 6). Left and right panels show two consecutive recordings separated by 300 s. Line shadows represent SEM. **g,** Percentage of reduction expressed as the minimum level activity observed after a wind-up plateau phase, 140 – 180 s; Paired Wilcoxon signed rank test (two-sided); ChR2 50.38 ± 8 vs dOVT 30.36 ± 5.46 %, W = -19, p = 0.0625, n_{cells} = 6 and **h** 570 – 600 s after BL onset. Paired Wilcoxon signed rank test (two-sided); ChR2 30.66 ± 7.78 vs dOVT 20.78 ± 9.41 %, W = -7 p = 0.5625, n_{cells} = 6.

i-n, vlPAG BL effect on the spike rate of WDR's Aβ-fiber discharge. **i,** Mean time course observed after vlPAG BL in control rats (gray, n = 8) and OT ChR2-expressing rats (blue, n = 14). Left and right panels show two consecutive recordings separated by 300 s. Line shadows represent SEM. **j** Percentage of reduction expressed as the minimum level activity observed after a wind-up plateau phase, 140 – 180 s; Unpaired Wilcoxon rank sum test (two-sided); Ctrl (n=8) 24.44 ± 10.14 vs ChR2 (n=15) 27.39 ± 7.91%, U = 55, p = 0.7763, and **k** 570 – 600 s after BL onset. Unpaired Wilcoxon rank sum test (two-sided); CTRL (n=8) 44.7 ± 13.23 vs ChR2 (n=14) 33.43 ± 10 %, U = 53, p = 0.8676. **l** Mean time course observed after vlPAG BL in OT ChR2-expressing rats (blue, n_{cells} = 6), and in the same recordings after dOVT injection in the vlPAG (red, n_{cells} = 6). Left and right panels show two consecutive recordings separated by 300 s. Line shadows represent SEM. **m** Percentage of reduction expressed as the minimum level activity observed after a wind-up plateau phase, 140 – 180 s; Paired Wilcoxon signed rank test (two-sided); ChR2 21.28 ± 10.25 vs dOVT 37.89 ± 8.12 %, W = 11, p = 0.3125, n = 6 and **n** 570 – 600 s after BL onset. Paired Wilcoxon signed rank test (two-sided); ChR2 20.14 ± 3.39 vs dOVT 34.58 ± 5.13 %, W = 17, p = 0.0938, n_{cells} = 6.

Results are expressed as the mean ± SEM and the individual points of each conditions are represented as white circle. Source data are provided as a Source Data file.

Figure S9

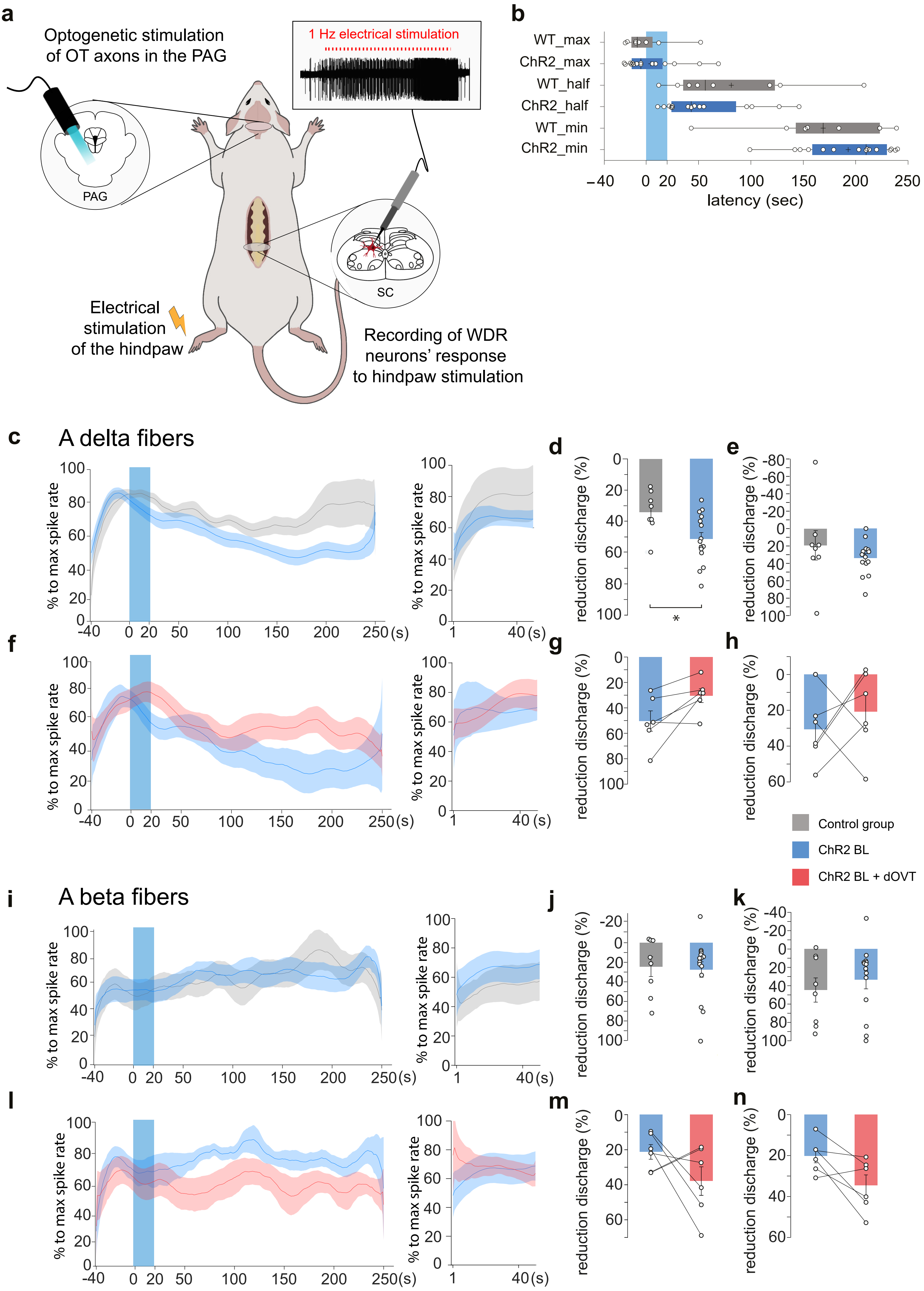
a
Optogenetic stimulation of OT axons in the PAG
PAG
1 Hz electrical stimulation
SC
Electrical stimulation of the hindpaw
Recording of WDR neurons' response to hindpaw stimulation
b
WT_max
ChR2_max
WT_half
ChR2_half
WT_min
ChR2_min
latency (sec)
c
A delta fibers
% to max spike rate
d
e
reduction discharge (%)
f
g
h
Control group
ChR2 BL
ChR2 BL + dOVT
i
A beta fibers
j
k
l
m
n

Supplementary Figure 10

a-c, Optogenetic stimulation of the vlPAG in the chronic constriction of the sciatic nerve (CCI) model of neuropathic pain in female rats. **a,** Schema of the injection of $rAAV_{1/2}$-OTp-ChR2-mCherry in the PVN and optic fiber implantation in the PAG. **b-c** Graphs showing the threshold response to mechanical stimuli in female rats optogenetically stimulated in vlPAG (as in Figure 6C and 6D). The effect of vlPAG-BL was measured at 5 min, 1 h and 3 h after vlPAG-BL on the CFA contralateral side (**b**) as well as CCI contralateral side (**c**). All results are expressed as average ± SEM. (**b**) 2-way RM ANOVA, $F_{treatment} = 8.391$; $p = 0.178$, $n_{rats} = 10$. (**c**) 2-way RM ANOVA, $F_{treatment} = 9.319$; $p = 0.2331$, $n_{rats} = 10$.

d-e, Verification for the expression of Gq-mCherry in the vlPAG. **d,** Schema of the injection of $rAAV_{1/2}$-EF1α-DIO-Gq-mCherry in the PAG. **e,** Image showing the expression of $rAAV_{1/2}$-EF1α-DIO-Gq-mCherry (red) with staining for DAPI (blue). Scale bar = 100 µm. Aq = aqueduct.

f-g, Thermal pain threshold of male rats expressing Gq-mCherry (red) or mCherry only (gray) in vlPAG OTR neurons after DCZ administration during normal or inflammation (CFA) conditions.

f, Mechanical pain threshold after DCZ administration in the CFA paw of male rats expressing Gq-mCherry (blue) or mCherry only (gray) in vlPAG OTR neurons. 2-way RM ANOVA test ($F_{interaction} = 17.79$; $p < 0.0001$; $n_{rats} = 6$), followed by multiple comparison post-hoc test with Dunnett correction: Gq-mCherry, 0 vs 20: $^{**}p_{adj} = 0.0033$. **g**, Thermal pain threshold after DCZ administration in the CFA paw of male rats expressing Gq-mCherry (red) or mCherry only (gray) in vlPAG OTR neurons. 2-way RM ANOVA test ($F_{interaction} = 40.40$; $p < 0.0001$; $n_{rats} = 6$), followed by multiple comparison post-hoc test with Dunnett correction: Gq-mCherry, 0 vs 20: $^{***}p_{adj} = 0.0003$; 0 vs 60: $^{**}p_{adj:} = 0.0077$; mCherry, 0 vs 180: $^{**}p = 0.0058$. Blue asterisks correspond to the statistic done on the Gq-mCherry group while black asterisks to the mCherry group.

h, Schema depicting the experimental timeline of the CPP protocol.

All results are expressed as average ± SEM. Source data are provided as a Source Data file.

Figure S10

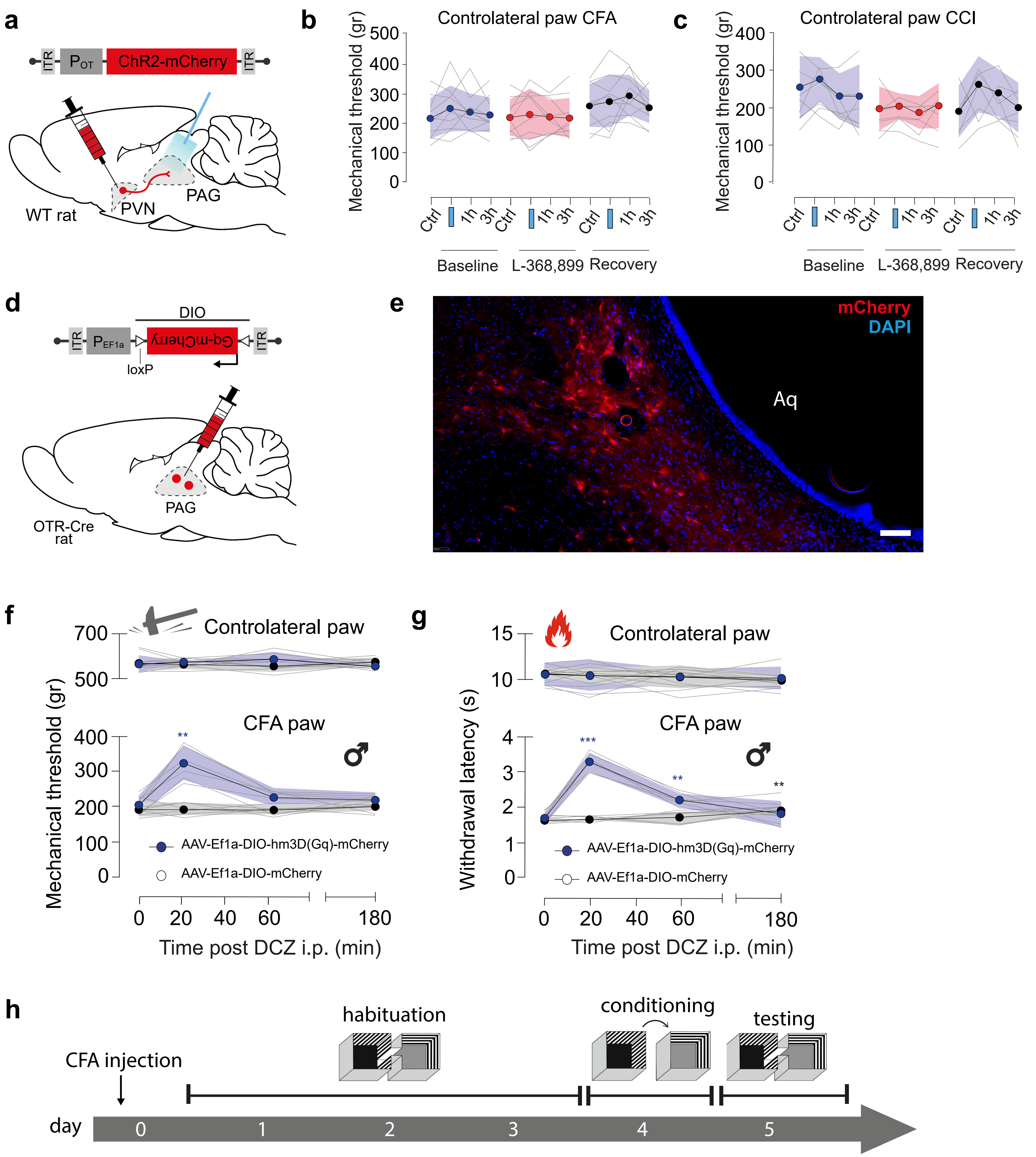

a
ITR
POT
ChR2-mCherry
ITR
WT rat
PVN
PAG
b
Controlateral paw CFA
Mechanical threshold (gr)
500
400
300
200
100
0
Ctrl
1h
3h
Baseline
L-368,899
Recovery
c
Controlateral paw CCI
Mechanical threshold (gr)
400
300
200
100
0
Baseline
L-368,899
Recovery
d
DIO
ITR
PEF1a
Gq-mCherry
ITR
loxP
OTR-Cre rat
PAG
e
mCherry
DAPI
Aq
f
Controlateral paw
CFA paw
Mechanical threshold (gr)
700
500
400
300
200
100
0
**
AAV-Ef1a-DIO-hm3D(Gq)-mCherry
AAV-Ef1a-DIO-mCherry
0
20
40
60
180
Time post DCZ i.p. (min)
g
Controlateral paw
CFA paw
Withdrawal latency (s)
15
10
4
3
2
1
0
***
**
**
AAV-Ef1a-DIO-hm3D(Gq)-mCherry
AAV-Ef1a-DIO-mCherry
0
20
40
60
180
Time post DCZ i.p. (min)
h
habituation
conditioning
testing
CFA injection
day
0
1
2
3
4
5
