# Supplementary material for: An analgesic pathway from parvocellular oxytocin neurons to the periaqueductal gray in rats
Source: Nat Commun. 2023 Feb 24;14:1066. doi: 10.1038/s41467-023-36641-7 (PMC9958129; doi:10.1038/s41467-023-36641-7)
Supplement: Supplementary file 3 — Reporting Summary [file 41467_2023_36641_MOESM3_ESM.pdf]

nature portfolio

Corresponding author(s): Alexandre Charlet

Last updated by author(s): Dec 30, 2022

Reporting Summary

Nature Portfolio wishes to improve the reproducibility of the work that we publish. This form provides structure for consistency and transparency in reporting. For further information on Nature Portfolio policies, see our Editorial Policies and the Editorial Policy Checklist.

Statistics

For all statistical analyses, confirm that the following items are present in the figure legend, table legend, main text, or Methods section.

n/a	Confirmed	
☐	☒	The exact sample size (*n*) for each experimental group/condition, given as a discrete number and unit of measurement
☐	☒	A statement on whether measurements were taken from distinct samples or whether the same sample was measured repeatedly
☐	☒	The statistical test(s) used AND whether they are one- or two-sided<br>*Only common tests should be described solely by name; describe more complex techniques in the Methods section.*
☐	☒	A description of all covariates tested
☐	☒	A description of any assumptions or corrections, such as tests of normality and adjustment for multiple comparisons
☐	☒	A full description of the statistical parameters including central tendency (e.g. means) or other basic estimates (e.g. regression coefficient) AND variation (e.g. standard deviation) or associated estimates of uncertainty (e.g. confidence intervals)
☐	☒	For null hypothesis testing, the test statistic (e.g. *F*, *t*, *r*) with confidence intervals, effect sizes, degrees of freedom and *P* value noted<br>*Give P values as exact values whenever suitable.*
☒	☐	For Bayesian analysis, information on the choice of priors and Markov chain Monte Carlo settings
☒	☐	For hierarchical and complex designs, identification of the appropriate level for tests and full reporting of outcomes
☐	☒	Estimates of effect sizes (e.g. Cohen's *d*, Pearson's *r*), indicating how they were calculated

Our web collection on statistics for biologists contains articles on many of the points above.

Software and code

Policy information about availability of computer code

Data collection	Anatomy data were collected using Excel 2010, IMARIS 9.8 and Zen 3.0 and 3.1. Ex vivo electrophysiological data were recorded using Clampex 10.7. Behavioral video acquisition were performed through Anymaze 7.1. In vivo electrophysiological data were recorded using MC Rack 4.6.2.
Data analysis	Anatomical data were analysed using Excel 2010, IMARIS 9.8 and Fiji. Electrophysiological data were analysed using Mini analysis 6.0, Graphpad Prism 7.05 and Matlab. Behavioral video analysis were performed through Anymaze 7.1. In vivo electrophysiological data were analysed using custom matlab script and wave_clus by Quian Quiroga. GRAB monitoring were analyzed through a lab made python code. Statistical analysis were performed using GraphPad Prism 7.05 and Matlab 9.1 R2016b. Python code for ex vivo GRAB analysis can be found at the following address: https://github.com/Etienneclcr/GRAB_PAG; Matlab code for in vivo electrophysiological recordings can be found at the following address: https://github.com/Etienneclcr/ephy-in-vivo-PAG.

For manuscripts utilizing custom algorithms or software that are central to the research but not yet described in published literature, software must be made available to editors and reviewers. We strongly encourage code deposition in a community repository (e.g. GitHub). See the Nature Portfolio guidelines for submitting code & software for further information.

Data

Policy information about availability of data

All manuscripts must include a data availability statement. This statement should provide the following information, where applicable:

- Accession codes, unique identifiers, or web links for publicly available datasets
- A description of any restrictions on data availability
- For clinical datasets or third party data, please ensure that the statement adheres to our policy

The raw data generated in this study have been deposited in the Zenodo database under accession code 10.5281/zenodo.7473865. The data generated in this study are provided in the Source Data file. In addition, all data that support the findings of this study are available from the corresponding authors upon request.

Human research participants

Policy information about studies involving human research participants and Sex and Gender in Research.

Reporting on sex and gender	not applicable
Population characteristics	not applicable
Recruitment	not applicable
Ethics oversight	not applicable

Note that full information on the approval of the study protocol must also be provided in the manuscript.

Field-specific reporting

Please select the one below that is the best fit for your research. If you are not sure, read the appropriate sections before making your selection.

[x] Life sciences [ ] Behavioural & social sciences [ ] Ecological, evolutionary & environmental sciences

For a reference copy of the document with all sections, see nature.com/documents/nr-reporting-summary-flat.pdf

Life sciences study design

All studies must disclose on these points even when the disclosure is negative.

Sample size	Based on our experience in the field, we used sample sizes similar to our previous studies (Eliava et al. 2016).
Data exclusions	No data was excluded.
Replication	All individual observations (Cre expression, anatomical qualitative observations) were repeated at least 5 times.
Randomization	Rats were randomly distributed to different groups. This was done by selecting subjects based on their inhouse number, to avoid selection bias.
Blinding	Due to the high level of experimental complexity, experiments were not performed blind.

Reporting for specific materials, systems and methods

We require information from authors about some types of materials, experimental systems and methods used in many studies. Here, indicate whether each material, system or method listed is relevant to your study. If you are not sure if a list item applies to your research, read the appropriate section before selecting a response.

Materials & experimental systems

n/a	Involved in the study	
☐	☒	Antibodies
☐	☒	Eukaryotic cell lines
☒	☐	Palaeontology and archaeology
☐	☒	Animals and other organisms
☒	☐	Clinical data
☒	☐	Dual use research of concern

Methods

n/a	Involved in the study	
☒	☐	ChIP-seq
☒	☐	Flow cytometry
☒	☐	MRI-based neuroimaging

Antibodies

Antibodies used

Chicken anti-GFP primary antibody Abcam ab13970
Mouse NeuN primary antibody Millipore MAB377
Rabbit vGluT2 primary antibody Synaptic systems 135 103
Rabbit anti-dsRed primary antibody Living Colors 632496
GAD67 Millipore MAB5406
Guinea-pig anti-Fluorogold primary antibody Protos Biotech Corp NM-101
Mouse c-fos polyclonal primary antibody Santa-Cruz sc-8047
Mouse monoclonal anti-OT primary antibody Provided by Dr. Harold Gainer PS 38
DAPI Vector Laboratories H-1200-10
Synaptophysin Abcam Ab32127
Rabbit polyclonal anti-vGluT2 primary antibody SYSY 135403

Validation

All antibodies were KO tested by the respective companies.

Eukaryotic cell lines

Policy information about cell lines and Sex and Gender in Research

Cell line source(s)

Human embryonic immortalized cell line HEK 293, taken from female fetus in 1973 (doi:10.1186/1471-2121-12-23)

Authentication

Morphological analysis, Quantification of HEK 293 DNA by qPCR

Mycoplasma contamination

None

Commonly misidentified lines
(See ICLAC register)

None

Animals and other research organisms

Policy information about studies involving animals; ARRIVE guidelines recommended for reporting animal research, and Sex and Gender in Research

Laboratory animals

We used both males and females rats aged between 8 and 20 weeks
Rattus Norvegicus (Sprague Dawleys) Charles River N/A
OTR-IRES-Cre (Sprague Dawleys) Lab made N/A

Wild animals

The study did not involved wild animals.

Reporting on sex

We took into account potential sex differences. Therefore, while most of the data were obtained in female rats, key experiments were reproduced in male rats, as stated in the manuscript.

Field-collected samples

The study did not involved field collected samples.

Ethics oversight

All experiments were conducted in accordance with European law, under French h Ministry of Higher Education and Research license 3668-2016011815445431 and 15541-2018061412017327, and German Animal Ethics Committee of the Baden Württemberg license G-102/17

Note that full information on the approval of the study protocol must also be provided in the manuscript.
